# Identification of missing hierarchical relations in the vaccine ontology using acquired term pairs

**DOI:** 10.1186/s13326-022-00276-2

**Published:** 2022-08-13

**Authors:** Warren Manuel, Rashmie Abeysinghe, Yongqun He, Cui Tao, Licong Cui

**Affiliations:** 1 School of Biomedical Informatics, The University of Texas Health Science Center at Houston, Houston, TX USA; 2 Department of Neurology, The University of Texas Health Science Center at Houston, Houston, TX USA; 3 Unit for Laboratory Animal Medicine, Department of Microbiology and Immunology, Center for Computational Medicine and Bioinformatics, University of Michigan Medical School, Ann Arbor, MI USA

**Keywords:** Vaccine ontology, Ontology quality assurance, Hierarchical relations

## Abstract

**Background:**

The Vaccine Ontology (VO) is a biomedical ontology that standardizes vaccine annotation. Errors in VO will affect a multitude of applications that it is being used in. Quality assurance of VO is imperative to ensure that it provides accurate domain knowledge to these downstream tasks. Manual review to identify and fix quality issues (such as missing hierarchical is-a relations) is challenging given the complexity of the ontology. Automated approaches are highly desirable to facilitate the quality assurance of VO.

**Methods:**

We developed an automated lexical approach that identifies potentially missing *is-a* relations in VO. First, we construct two types of VO concept-pairs: (1) linked; and (2) unlinked. Each concept-pair further derives an Acquired Term Pair (ATP) based on their lexical features. If the same ATP is obtained by a linked concept-pair and an unlinked concept-pair, this is considered to indicate a potentially missing *is-a* relation between the unlinked pair of concepts.

**Results:**

Applying this approach on the 1.1.192 version of VO, we were able to identify 232 potentially missing *is-a* relations. A manual review by a VO domain expert on a random sample of 70 potentially missing *is-a* relations revealed that 65 of the cases were valid missing *is-a* relations in VO (a precision of 92.86%).

**Conclusions:**

The results indicate that our approach is highly effective in identifying missing *is-a* relation in VO.

## Background

A vaccine as defined by the Centers for Disease Control and Prevention (CDC) is a preparation that is used to stimulate the body’s immune response against diseases and is usually administered via needles while oral and nasal sprays are also available [1]. Vaccines have been able to create a transformation in public health and have since been able to prevent between 2-3 million deaths annually from diseases such as diphtheria, tetanus, influenza, and measles; showing a reduction of under-five mortality globally from 93 to 38 deaths per 1000 live deaths in the time span from 1990 to 2018 [2]. Especially during the recent COVID-19 pandemic, vaccine research and development have become more important than ever.

Due to the clinical usage and extensive research regarding vaccines, it has become necessary to standardize vaccine annotation, combine vaccine information from disparate sources, and support machine-readability. To address such challenges, the Vaccine Ontology (VO) has been developed [3, 4]. VO focuses on vaccine categorization, vaccine components, vaccine quality, and vaccine-induced host responses [5]. The core terms in VO span from the vaccine to the pathogen, the vaccine’s administration and its immune response. The 1.1.192 version of VO contains 6,883 concepts. VO has reused terms from external ontologies such as Chemical Entities of Biological Interest (CHEBI) [6], Foundational Model of Anatomy (FMA) [7], and Infectious Disease Ontology (IDO) [8] supporting ontology interoperability.

Being a community-based ontology in the domain of rapidly evolving biomedical knowledge, VO may suffer from incomplete knowledge and inconsistent modelling. For example, “*infectious bursal disease virus vaccine*” (with VO concept identifier VO:0001497) is claimed as a subconcept of “*viral vaccine*” (VO:0000609), but “*live attenuated infectious bursal disease virus vaccine*” (VO:0000961) is not claimed as a subconcept of “*live attenuated viral vaccine*” (VO:0001220). It is imperative that VO is audited so that quality issues can be identified and addressed especially because it has been used in a multitude of applications including vaccine data integration [9–12] and literature mining systems [13, 14]. Such applications may be less accurate due to the propagation of quality issues from VO.

Ontology development tools such as Protégé [15, 16] ensure the syntactical accuracy of an ontology by providing inbuilt reasoning support for identifying implicit subsumptions and logical inconsistencies. However, this could be of limited value in quality assurance as ontologies may contain missing information. Errors of omission would rarely lead to logical inconsistencies being caught by such methods [17]. Identifying such issues through manual inspection by domain experts is a challenging task. Although VO is a rather small ontology with 6,882 concepts when compared to other biomedical ontologies such as Gene Ontology (GO) [18–20] (43,699 in the 2022-03 release) and SNOMED CT [21] (498,686 concepts in the 2022-03 US release), manual review to identify quality issues is neither practical nor sustainable. Therefore, it is important to explore automated or semi-automated methods to aid in the identification of potential quality issues.

The principal objective of automated and semi-automated methods for quality analysis of biomedical ontologies is to uncover concepts with high likelihood of being problematic, which can then be reviewed by a subject domain expert for verification. Various approaches have been investigated for identifying different quality issues of biomedical ontologies [22, 23]. For instance, abstraction networks have been widely investigated for quality analysis of biomedical ontologies [24]. Abstraction networks are a type of summary graphs of an ontology providing a higher level view of its content, where nodes within the abstraction network summarized similar concepts within the ontology based on relationships. As an example, Min et al. [25] have applied abstraction networks on the National Cancer Institute thesaurus (NCIt) to discover potentially erroneous concepts, which were further examined by human reviewers to identify specific quality issues including missing roles, missing concepts, and incorrect IS-A relations. Quesada-Martinez et al. [26] have investigated the correspondence between the content in natural language in a concept label with the logical axioms of the concept to uncover missing relations in SNOMED CT. Rector et al. [27] have investigated the SNOMED CT expressions for acute and chronic findings. They have compared the concepts lexically as well as semantically which has lead to the identification of certain modelling irregularities. In previous work, we have explored non-lattice subgraphs to identify missing *is-a* relations and missing concepts in SNOMED CT, NCIt, and GO [28–33]. Non-lattice subgraphs indicate ontology fragments that violate lattice-property, a desirable structural indicator for a well-formed ontology [34]. Additionally, we have investigated a lexical-based inference approach to explore lexical irregularities between GO concept-pairs with and without *is-a* relations [35, 36]. To our knowledge, such systematic approaches targeted to auditing VO have not been studied in prior work. Therefore, in this work, we introduce an automated lexical approach to uncover potentially missing *is-a* relations in VO.

## Methods

To demonstrate our approach, we used the 1.1.192 version (released on 03/19/2022) of VO in Web Ontology Language (OWL). Utilizing the OWLReady2 python library [37], we obtain the names and ancestors of each VO concept. Then, leveraging the ancestor information obtained, we generate linked and unlinked-pairs of concepts. Each pair of concepts with common lexical feature(s) will further derive an acquired term pair (ATP) denoting the term difference between the two concepts. If the same ATP can be obtained by both a linked concept-pair and an unlinked concept-pair, then the unlinked concept-pair is flagged as indicating a potentially missing *is-a* relation.

### Representation of concepts

Our approach requires a concept to be represented as a set of its features. In this work, we obtain the features from concept names as follows. We first convert the name of a concept to lowercase. Then we tokenize the concept name to words and remove duplicate words. The result would be a set of words which can be considered as the lexical features corresponding to the name of the concept. For example, consider the concept “*Hepatitis B Surface Antigen Vaccine 0.01 MG/ML*” (VO:0003423). This concept would be represented as {*hepatitis, b, surface, antigen, vaccine, 0.01, mg/ml*}.

### Generation of linked concept-pairs

We leverage the ancestor information for each concept obtained through OWLReady2 to construct a set of linked concept-pairs as follows. A given concept-pair *C* and *A* would form a linked concept-pair *L*(*C,A*) if the following constraints are satisfied:

1. if *A* is an ancestor of *C*; and
2. if *C* and *A* have at least a single common lexical feature.

Note that linked concept-pairs are ordered pairs. That is, *L*(*C,A*) indicate that *C* is the descendant and *A* is the ancestor. This means that *L*(*C,A*) and *L*(*A,C*) are different pairs. However, usually *L*(*C,A*) and *L*(*A,C*) would not both exist in an ontology as they would form a cycle.

**Fig. 1:**
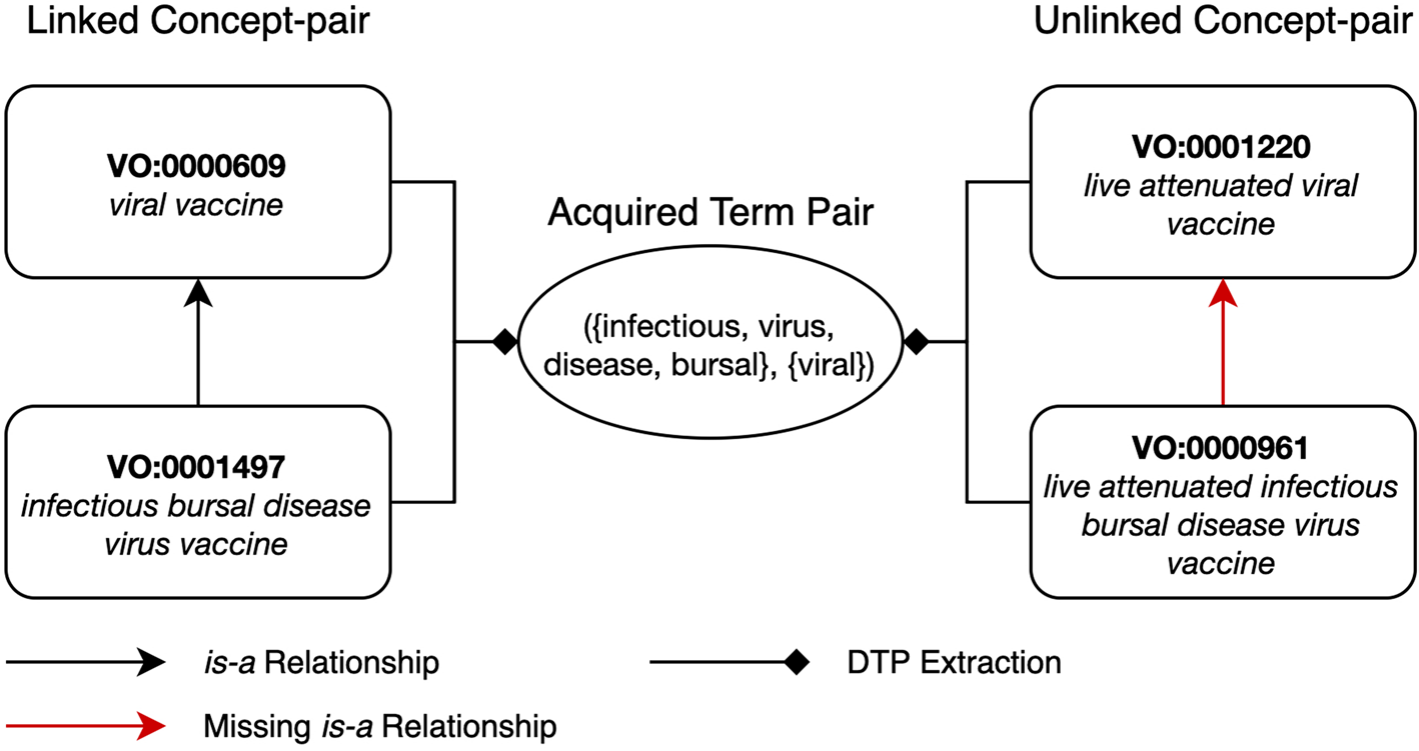
Valid missing *is-a* suggestion between concepts VO:0000961 and VO:0001220. A missing *is-a* relation identified between the concepts “*live attenuated infectious bursal disease virus vaccine*” (VO:0000961) and “*live attenuated viral vaccine*” (VO:0001220) was confirmed as valid by the domain expert

**Fig. 2:**
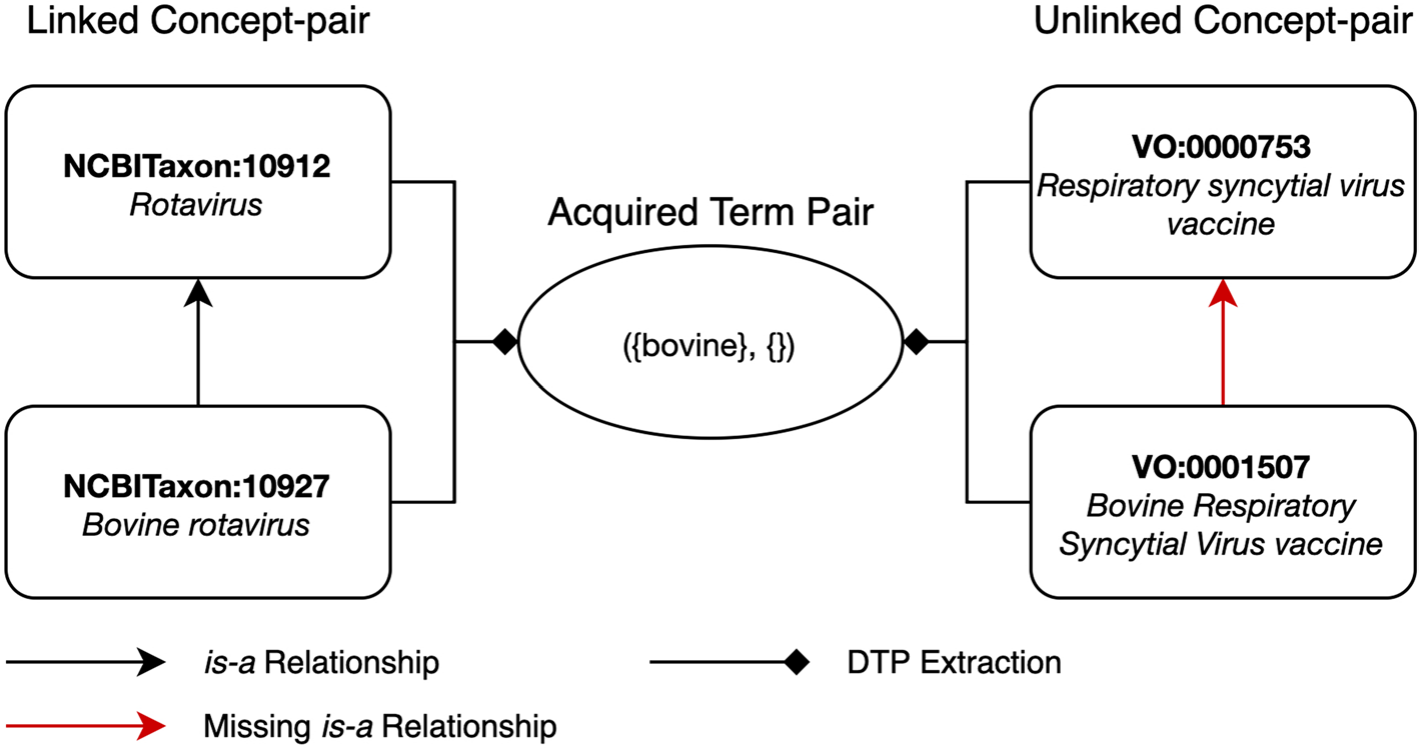
Valid missing *is-a* relation between concepts VO:0001507 and VO:0000753. A missing *is-a* relation identified between the concepts “*Bovine Respiratory Syncytial Virus vaccine*” (VO:0001507) and “*Respiratory syncytial virus vaccine*” (VO:0000753) was confirmed as valid by the domain expert

For example, the concepts “*infectious bursal disease virus vaccine*” (VO:0001497) and “*viral vaccine*” (VO:0000609) in Fig. 1 form a linked concept-pair as VO:0000609 is the parent of VO:0001497 and both the concepts have the common lexical feature: {*vaccine*}. Similarly, considering Fig. 2, the concepts “*Bovine rotavirus*” (NCBITaxon:10927) and “*Rotavirus*” (NCBITaxon:10912) form a linked concept-pair.

We iterate through ancestors of all the concepts and construct a set of all linked concept-pairs.

### Generation of unlinked concept-pairs

We leverage the hierarchical information of VO to construct a set of unlinked concept-pairs. A given concept-pair *C* and *D* would form an unlinked concept-pair *U*(*C,D*) only if the following conditions are satisfied:

1. if *C*≠*D*;
2. if *D* is not an ancestor of *C* and *C* is not an ancestor of *D*;
3. if *C* and *D* have at least a single common lexical feature;
4. if *C* and *D* both belong to the same ontology (note that VO contains external ontology concepts); and
5. if *C* and *D* fall within the same subhierarchy out of the 19 different subhierarchies under concept “*material entity*” (BFO:0000040) of VO.

Here the fifth condition requires the unlinked concept-pair to be in the same subhierarchy of “*material entity*” for the following reasons: (1) the vast majority of VO concepts (including vaccines) are under “*material entity*”; and (2) the subhierarchies under “*material entity*” model different domains of VO.

Note that unlinked concept-pairs are ordered as well. That is, *U*(*C,D*) is considered to be different from *U*(*D,C*). However, in certain situations, one of them could form a linked concept-pair. For instance, if *U*(*C,D*) is an unlinked concept-pair but *L*(*D,C*) is a linked concept-pair, then we do not include *U*(*C,D*) in the unlinked concept-pair set. Otherwise, both are included.

As an example, the concepts “*live attenuated infectious bursal disease virus vaccine*” (VO:0000961) and “*live attenuated viral vaccine*” (VO:0001220) in Fig. 1 form an unlinked concept-pair as VO:0001220 is not an ancestor of VO:0000961, both the concepts are in the subhierarchy rooted under “*processed material*” (OBI:0000047) which is a subhierarchy under “*material entity*” (BFO:0000040) and both the concepts have common lexical features {*live, attenuated, vaccine*}. Similarly, in Fig. 2, the concepts “*Bovine Respiratory Syncytial Virus vaccine*” (VO:0001507) and “*Respiratory syncytial virus vaccine*” (VO:0000753) form an unlinked-pair.

For each subhierarchy under “*material entity*” (BFO:0000040), we iterate through all combinations of concept-pairs and construct a set of all unlinked concept-pairs.

### Generation of acquired term pairs

A linked or unlinked concept-pair derives an ATP which emphasises the unique lexical features of each concept. Let the lexical features of a concept-pair *C*_1_ and *C*_2_ be *F*(*C*_1_) and *F*(*C*_2_) respectively. Then the ATP generated by the concepts is defined as:

$$ATP(C_{1}, C_{2}) = ({F(C_{1})-F(C_{2})}, {F(C_{2})-F(C_{1})}),$$

i.e., the ATP is obtained by removing common lexical features and maintaining unique ones. For instance, consider the linked concept-pair “*infectious bursal disease virus vaccine*” (VO:0001497) and “*viral vaccine*” (VO:0000609) in Fig. 1. By removing common lexical features, we obtain ({infectious, bursal, disease, virus}, {viral}) as the ATP. Similarly, from the unlinked concept-pair “*Bovine Respiratory Syncytial Virus vaccine*” (VO:0001507) and “*Respiratory syncytial virus vaccine*” (VO:0000753) in Fig. 2, we obtain the ATP ({bovine}, {}). The second set of the ATP in this instance is an empty set since the lexical features of concept VO:0000753 form a subset of the lexical features of VO:0001507.

Note that a concept-pair (*C*_1_,*C*_2_) would generate an *ATP*(*C*_1_,*C*_2_) that is different from an *ATP*(*C*_2_,*C*_1_) generated by concept-pair (*C*_2_,*C*_1_).

### Discovery of potentially missing *is-a* relations

Given a linked concept-pair *L*(*C*_1_,*C*_2_) and an unlinked concept-pair *U*(*C*_3_,*C*_4_), if *ATP*(*C*_1_,*C*_2_)=*ATP*(*C*_3_,*C*_4_), then we suggest a potentially missing *is-a* relation: *C*_3_*is-a* *C*_4_. In other words, if an ATP derived by a linked concept-pair can also be derived by an unlinked concept-pair, this is considered to indicate a potentially missing *is-a* relation among the unlinked concept-pair.

For example, in Fig. 1, the linked concept-pair “*infectious bursal disease virus vaccine*” (VO:0001497) and “*viral vaccine*” (VO:0000609) derive the ATP ({infectious, virus, disease, bursal}, {viral}), which can also be derived by the unlinked concept-pair “*live attenuated infectious bursal disease virus vaccine*” (VO:0000961) and “*live attenuated viral vaccine*” (VO:0001220). Hence, this denotes a potentially missing *is-a* relation: VO:0000961 *is-a* VO:0001220.

Similarly, in Fig. 2, the linked concept-pair “*Bovine rotavirus*” (NCBITaxon:10927) and “*Rotavirus*” (NCBITaxon:10912) as well as the unlinked concept-pair “*Bovine Respiratory Syncytial Virus vaccine*” (VO:0001507) and “*Respiratory syncytial virus vaccine*” (VO:0000753), derive the same ATP ({bovine}, {}), and thus indicate a potentially missing *is-a* relation: VO:0001507 *is-a* VO:0000753. Note that as in this example, the linked or unlinked concept-pairs may originate from external ontologies as VO reuses concepts from external ontologies.

Given the unlinked-pairs and linked-pairs, Algorithm 1 shows the procedure that was used to extract such potentially missing *is-a* relations.

Note that the same potentially missing *is-a* relation *C*_3_*is-a* *C*_4_ may be obtained leveraging multiple linked concept-pairs *L*(*C*_1_,*C*_2_) and *L*(*C*_5_,*C*_6_) if they derive the same ATP. We remove such duplicate cases from our final set of potentially missing *is-a* relations.

### Post-processing

We further perform a filtration step on the set of potentially missing *is-a* relations as described below. For an unlinked concept-pair *U*(*C*_3_,*C*_4_) and a linked concept-pair *L*(*C*_1_,*C*_2_) generating the same *ATP*, if the name of the concept *C*_3_ is the same as the concept *C*_1_ or the name of the concept *C*_4_ is the same as the concept *C*_2_, then we do not suggest a potentially missing *is-a* relation between *C*_3_ and *C*_4_. This is because two concepts with the same name but different identifiers may reveal a different type of quality issues (e.g., duplicate concepts) rather than a missing *is-a* relation. For example, the linked concept-pair “*F fusion protein*” (VO:0011167) and “*Measles virus protein*” (VO:0010784) and the unlinked concept-pair “*F fusion protein*” (VO:0011208) and “*Measles virus protein*” (VO:0010784) generate the same ATP: ({f, fusion}, {measles, virus}). However, VO:0011167 and VO:0011208 have the same name: “*F fusion protein*”. Hence, we do not suggest a missing *is-a* relation between VO:0011208 and VO:0010784.


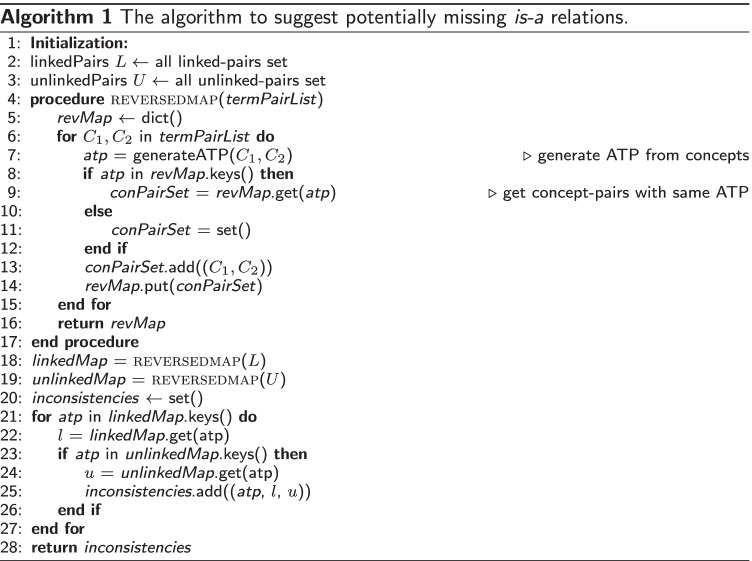


### Manual evaluation of identified potentially missing *is-a* relations

Potentially missing *is-a* relations identified by this method need to be manually reviewed for validation and confirmation before their adoption to VO. We randomly selected a subset of suggested potentially missing *is-a* relations for domain expert evaluation. For each missing *is-a* relation in the subset, the names of the two concepts together with their identifiers were provided to a domain expert (author YH, who has expertise in microbiology, vaccinology, and nephrology, and currently leads the development of VO). The domain expert examined whether the suggested relation is valid; not only theoretically, but also in terms of its suitability to current modeling practices of VO.

## Results

Table 1 displays the summary statistics of our study. In total the method identified 232 potentially missing *is-a* relations in VO. Table 2 shows 10 examples of valid missing *is-a* relations identified by our approach, as well as their ATPs and corresponding linked concept-pairs. As an example, our method suggested that “*inactivated acellular pertussis vaccine*” (VO:0003196) is a descendant of “*inactivated vaccine*” (VO:0000315). The 232 potentially missing relations were based on 120 distinct ATPs. Table 3 shows the 10 ATPs which generated the highest number of potentially missing *is-a* relations. For instance, the ATP ({quadrivalent},{}) accounted for 12.07% (28/232) potentially missing *is-a* relations.

**Table 1:** Summary Statistics

Total concepts	6,883
Linked concept-pairs	62,538
Unlinked concept-pairs	17,301,802
Linked concept-pairs generating ATPs	15,470
Unlinked concept-pairs generating ATPs	8,659,034
Potentially missing *is-a* relations	232

**Table 2:** Ten examples of valid missing *is-a* relations

Missing *is-a* Relations (Unlinked concept-pair)	ATP	Linked concept-pair
*inactivated acellular pertussis vaccine* (VO:0003196)	{acellular, pertussis},{}	*Acellular Pertussis Vaccine* (VO:0003389)
*inactivated vaccine* (VO:0000315)		*vaccine* (VO:0000001)
*Gardasil 9 prefilled syringe* (VO:0015038)	{syringe, prefilled},{injectable, product}	*Hepatitis B Surface Antigen Vaccine Prefilled Syringe* (VO:0003419)
*Gardasil 9 Injectable Product* (VO:0015039)		*Hepatitis B Surface Antigen Vaccine Injectable Product* (VO:0003415)
*inactivated avian influenza vaccine* (VO:0001024)	{avian, influenza},{viral}	*avian influenza vaccine* (VO:0000461)
*inactivated viral vaccine* (VO:0000712)		*viral vaccine* (VO:0000609)
*COVID-19 recombinant vector vaccine* (VO:0005199)	{covid-19},{viral}	*COVID-19 vaccine* (VO:0004908)
*recombinant viral vector vaccine* (VO:0005331)		*viral vaccine* (VO:0000609)
*Hepatitis A Vaccine, Inactivated* (VO:0003464)	{hepatitis, a},{}	*Hepatitis A virus protein* (VO:0010780)
*inactivated vaccine* (VO:0000315)		*virus protein* (VO:0010754)
*Acellular Pertussis Vaccine* (VO:0003389)	{pertussis},{}	*Bordetella pertussis* (NCBITaxon:520)
*acellular vaccine* (VO:0000756)		*Bordetella* (NCBITaxon:517)
*COVID-19 RNA vaccine* (VO:0005198)	{covid-19},{}	*COVID-19 vaccine* (VO:0004908)
*RNA vaccine* (VO:0000186)		*vaccine* (VO:0000001)
*acellular pertussis vaccine, inactivated* (VO:0003390)	{inactivated, acellular, pertussis},{bordetella}	*inactivated acellular pertussis vaccine* (VO:0003196)
*Bordetella vaccine* (VO:0000587)		*Bordetella vaccine* (VO:0000587)
*Human papillomavirus protein* (VO:0010786)	{papillomavirus},{}	*Papillomavirus vaccine* (VO:0000748)
*human protein* (VO:0000516)		*vaccine* (VO:0000001)
*licensed influenza vaccine* (VO:0003143)	{influenza},{}	*Influenza virus protein* (VO:0010782)
*licensed vaccine* (VO:0000363)		*virus protein* (VO:0010754)

**Table 3:** Ten ATPs that generated the most potentially missing *is-a* relations

ATP	No. of potentially missing *is-a* relations
{quadrivalent},{}	28
{ml, injection, 0.5},{}	13
{licensed},{}	9
{ml, syringe, 0.5, prefilled},{}	8
{1, ml, injection},{}	7
{covid-19},{}	7
{1, ml, syringe, prefilled},{}	5
{abortus, brucella},{}	5
{authorized, covid-19},{}	5
{injectable, product},{}	5

### Evaluation

To assess the effectiveness of our approach in discovering valid missing *is-a* relations, we constructed a random sample of 70 potentially missing *is-a* relations for manual evaluation by the VO domain expert. The 70 cases were selected so that no two cases correspond to the same ATP to avoid reviewing similar cases. The evaluation revealed that 65 of our suggestions represent valid missing *is-a* relations in VO. Therefore, the overall precision of the method is 92.86% (65/70).

## Discussion

In this work, we investigated a lexical approach to extract *is-a* relation inconsistencies from the Vaccine Ontology. We constructed two types of concept-pairs: linked and unlinked. For the unlinked concept-pairs, we focused on the different subhierarchies under the “*material entity*” (BFO:0000040) concept. This is because we needed to pair unrelated but relevant concepts as unlinked concept-pairs. Each subhierarchy under “*material entity*” (BFO:0000040) models different domains such as “*anatomical entity*”, “*antigen*”, “*chemical entity*”, “*gene*”, etc. Therefore, concepts under each of these subhierarchies have a certain degree of relevance with each other. This is important as pairing irrelevant concepts may not only lead to the extraction of invalid missing *is-a* relations, but would also increase the running time of the method. In fact, VO currently does not have any *is-a* relations across these subhierarchies. It should also be noted that out of the 6,883 concepts in the 1.1.192 version of VO used in this work, 5,672 concepts (82.4%) were in these subhierarchies.

### Analysis of ATP size

**Fig. 3:**
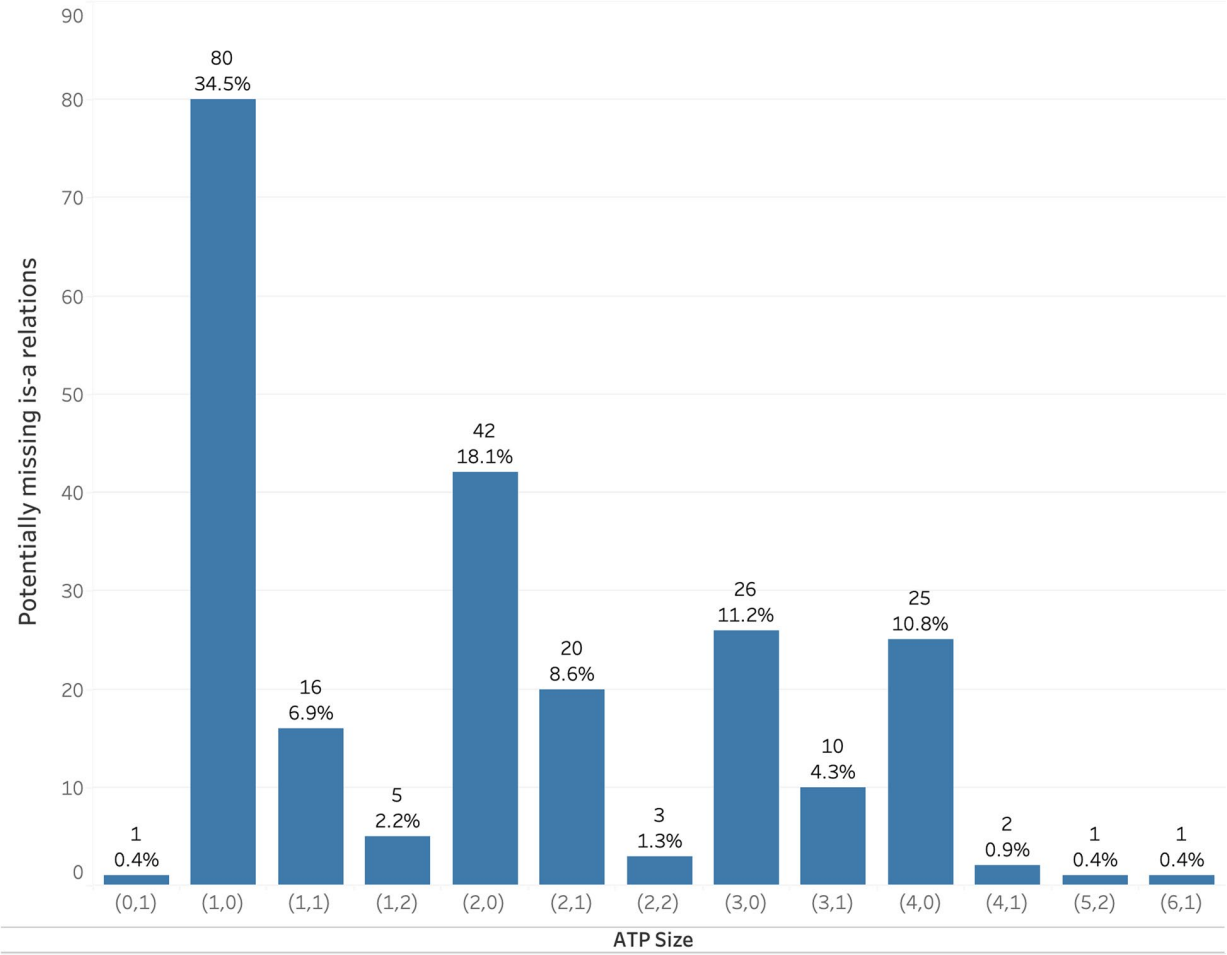
ATP size distribution of potentially missing *is-a* relations in VO

We denote the size of an ATP by a pair of integers corresponding to the sizes of the two sets the ATP contains. For instance, the size of the ATP ({inactivated, acellular, pertussis}, {bordetella}) is (3, 1). Figure 3 shows the distribution of the ATP sizes across the 232 potentially missing *is-a* relations uncovered in this work. For example, 80 missing *is-a* relation suggestions had ATPs of size (1, 0), while only 16 had size (1, 1). The analysis of the ATP sizes as displayed in Fig. 3 highlights that the majority of the potential *is-a* relations (74.57%) correlate with situations where the second set in the ATP is an empty set. This happens when the lexical features of the potential ancestor form a subset of the lexical features of the potential descendant. In fact, the most number of *is-a* relations were suggested by the following ATP size: (1, 0), (2, 0), (3, 0), and (4, 0).

In addition, it can be seen that for a certain size of the first set of the ATP, when the size of the second set increases, the number of suggested missing *is-a* relations decreases. For instance, (1, 0) was observed in 80 potentially missing *is-a* relations, while (1, 1) was observed in 16, and (1, 2) was observed in 5. Since the ATP contains unique words in each concept, this shows that in a majority of missing *is-a* relations, more unique words can be found on the descendant concept than the ancestor concept. This is expected as descendant concepts are supposed to be more specific concepts than ancestors.

### Analysis of false positives

**Fig. 4:**
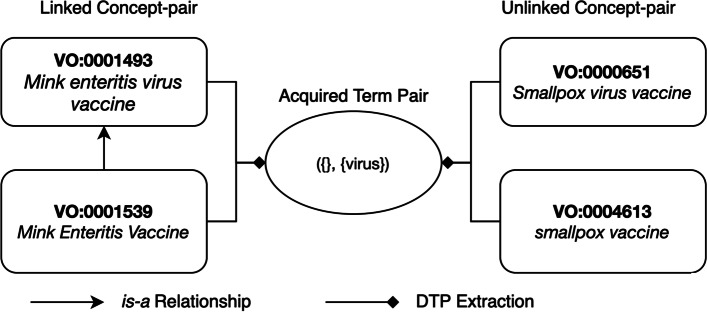
Invalid missing *is-a* relation suggestion between concepts VO:0004613 and VO:0000651. A missing *is-a* relation identified between concepts “*smallpox vaccine*” (VO:0004613) and “*Smallpox virus vaccine*” (VO:0000651) was found to be invalid by the domain expert

Based on the evaluation by the VO domain expert, it was seen that 5 of our missing *is-a* suggestions were invalid. For example, as displayed in Fig. 4, our approach suggested an invalid missing *is-a* relation between concepts “*smallpox vaccine*” (VO:0004613) and “*Smallpox virus vaccine*” (VO:0000651). However, these concepts were found to be synonyms that need to be merged into one concept. Therefore, in this instance, while our suggestion is invalid, it has lead into the identification of a different inconsistency in VO.

In another example, our method suggested a potentially missing *is-a* relation between concepts “*Brucella canis*” (NCBITaxon:36855) and “*Canis*” (NCBITaxon:9611), which was found to be invalid. This is because brucella canis is a bacterium, while canis is a mammal. Our method also suggested an invalid missing *is-a* relation between concepts “*Corynebacterium pseudotuberculosis*” (NCBITaxon:1719) and “*Corynebacterium diphtheriae*” (NCBITaxon:1717). These terms are actually siblings under the parent concept “*Corynebacterium*” (NCBITaxon:144193) and do not form a valid *is-a* relation. Note that these two false positives were missing *is-a* suggestions among concept-pairs in the NCBI Taxonomy [38], an external ontology. If a missing *is-a* suggestion for an external ontology was valid, the actual fix would need to be handled by the external ontology so that VO could re-import and re-use the corrected version.

The expert was indecisive about two of the missing *is-a* suggestions. Regarding the suggested *is-a* relation between “*Varicella-Zoster Virus Vaccine Live (Oka-Merck) strain 29800 UNT/ML*” (VO:0003279) and “*Varicella-Zoster Virus Vaccine Live (Oka-Merck) strain Injection*” (VO:0003274), the expert was uncertain whether the former concept always corresponds to an injection. For the suggested *is-a* relation between concepts “*toxoid vaccine*” (VO:0000561) and “*toxoid*” (VO:0001252), the expert was uncertain about the validity as the latter concept corresponds to a component of the former. We categorized both these cases as false positives.

### Comparison with related work

A major distinction between our approach and most other ontology quality assurance approaches is its capability of identifying the exact quality issues, as a result of which less manual review effort is needed by domain experts. For example, abstraction networks based approaches [24, 25] only identify problematic areas of ontologies requiring considerable effort from domain experts to manually uncover the exact quality issues. In contrast, our approach merely requires experts to validate whether the exact quality issues suggested are valid or not. Additionally, to the best of our knowledge, there has no prior work on investigation of quality assurance approaches for VO.

Note that due to the discovery nature of the ontology quality assurance task [39], different approaches may have revealed distinct instances of quality issues that have not been discovered previously. In addition, there is a lack of benchmark datasets that can be used for fairly comparing the performance of different quality assurance approaches.

In prior work, we introduced a similar approach leveraging word differences between concepts to audit the Gene Ontology [35, 36]. However, the criteria for the selection of concept-pairs were different than what was used in this work. In the prior work, the concept-pair was limited to have the same number of words and *n* different words (only *n*=1,2,3,4,5 were considered). In this work, we generalized the approach without such limitations, and applied it to the Vaccine Ontology, for which according to our knowledge, systematic automated approaches have not being developed for auditing purposes. Additional improvement of the approach in this work is requiring the unlinked concept-pair to be within the same subhierarchy, which was not leveraged in the prior work and without which invalid cross-hierarchical missing *is-a* relations would be more likely to be suggested.

### Limitations and future directions

One limitation of this work is that our concepts were represented by lexical features obtained from concept names. An interesting future direction is to investigate whether incorporating additional attributes of concepts such as other lexical metadata (e.g., synonyms), ancestor lexical features, attribute relations, etc. would improve the overall results in terms of the number of potentially missing *is-a* discovered and the precision of the method. The features may also be imported from mapped concepts in external ontologies. Additionally, the only normalization that was performed in this work was to convert the lexical features to lowercase. Leveraging other strategies like lemmatization and synonym identification may help identify additional potentially missing *is-a* relations.

Another limitation of the method is that the missing *is-a* relations identified by this method are only between concept-pairs with common words. In the future, we will explore other strategies that can also handle cases between concepts without any common words. A particularly interesting direction is to investigate whether machine learning could be of help. To train a machine learning model, the lexical features of concepts will need to be represented numerically using techniques such as word embedding. These representations would embed the meaning of the lexical features and therefore, through machine learning it might be possible to learn complex relationships between lexical features of the concept-pairs. Hence, such an approach may have the potential to predict a missing *is-a* relation between any concept-pair, not restricting to concept-pairs with common words.

A limitation of our evaluation is that only a subset of the missing *is-a* suggestions were evaluated by one expert. The precision of the method may be different if the size of the evaluation samples varies or multiple experts were involved. However, it should be noted that our expert (YH) is currently leading the development of VO. Since only 70 out of 232 missing *is-a* suggestions were evaluated (30.17%), a future work would be getting the entire set of 232 evaluated and incorporated into a future release of VO.

## Conclusions

In this paper, we presented an automated lexical approach to discover potentially missing *is-a* relations in the Vaccine Ontology, by leveraging term differences between concept-pairs. A total of 232 potentially missing *is-a* relations were suggested by our approach applied on the 1.1.192 version of the ontology. A random sample of 70 potentially missing *is-a* relations was evaluated by a Vaccine Ontology domain expert and 65 of them were confirmed as valid cases. The results revealed that our lexical approach is highly effective in identifying missing *is-a* relations in the Vaccine Ontology.

## Data Availability

The code as well as the results (all results and evaluation sample) can be found at: https://github.com/warren-manuel/vo_quality_assurance.

## Abbreviations

ATP: Acquired Term Pair
CDC: Centers for Disease Control and Prevention
CHEBI: Chemical Entities of Biological Interest
FMA: Foundational Model of Anatomy
IDO: Infectious Disease Ontology
GO: Gene Ontology
NCIt: National Cancer Institute thesaurus
OWL: Web Ontology Language
VO: Vaccine Ontology
